# A retrospective, observational study of real-world clinical data from the Cognitive Function Development Therapy program

**DOI:** 10.3389/fnhum.2024.1508815

**Published:** 2024-12-18

**Authors:** Eric D. Kirby, Brian Beyst, Jen Beyst, Sonia M. Brodie, Ryan C. N. D’Arcy

**Affiliations:** ^1^BrainNet, Health and Technology District, Surrey, BC, Canada; ^2^Faculty of Individualized Interdisciplinary Studies, Simon Fraser University, Burnaby, BC, Canada; ^3^Faculty of Science, Simon Fraser University, Burnaby, BC, Canada; ^4^Centre for Neurology Studies, HealthTech Connex, Metro Vancouver, BC, Canada; ^5^Cognitive Function Development Institute, Prescott Valley, AZ, United States; ^6^Djavad Mowafaghian Centre for Brain Health, Faculty of Medicine, University of British Columbia, Vancouver, BC, Canada; ^7^Faculty of Applied Sciences, Simon Fraser University, Burnaby, BC, Canada

**Keywords:** primary cognitive functions, cognitive training, cognitive testing, EEG, ERPs, brain vital signs

## Abstract

**Introduction:**

Cognitive deficits are common in psychiatric and mental health disorders, making the assessment of cognitive function in mental health treatment an important area of research. Cognitive Function Development Therapy (CFDT) is a novel therapeutic modality designed to enhance cognitive function and regulate the autonomic nervous system through targeted exercises and activities focused on attention networks and memory systems. The therapy is tracked and based on Primary Cognitive Function (PCF) scores.

**Methods:**

This retrospective, observational study analyzed real world data from 183 children and adults undergoing CFDT to evaluate changes in cognition over time, incorporating both cognitive performance measures and an exploratory analysis of neurophysiological function. Objective neurophysiological measures in the form of the brain vital signs framework, based in event-related potentials (ERPs), were measured in a small subset of clients to explore the frameworks use in CFDT.

**Results:**

Our findings indicate that CFDT holds promise for improving cognitive performance, as evidenced by increased PCF scores at the group level compared to pre-treatment levels [*F* (5, 173) = 7.087, *p* < 0.001, η_p_^2^ = 0.170]. Additionally, a weak effect of age [Spearman’s Rho range: −0.301 to −0.340, *p* < 0.001] was found to influence the degree of cognitive improvement, suggesting the importance of early intervention for maximizing cognitive gains. The exploratory analysis suggested that CFDT may affect neurophysiological measures of information processing, particularly in basic attention, as reflected in increased amplitude in P300 measures.

**Discussion:**

While these initial findings are encouraging, caution is warranted due to the retrospective nature of the study, though overall, the results suggest a positive impact of CFDT on cognitive function.

## Introduction

Cognitive training aims to drive neuroplastic changes in neural systems, which can improve cognitive processes related to emotion regulation and clinical symptoms across various neuropsychiatric disorders (Haas et al., 2021; Thomas et al., 2018; Hochberger et al., 2019; Vinogradov et al., 2012). Unfortunately, traditional therapeutic modalities that address distorted cognitions, focus on emotional expression, or expose individuals to traumatic memories, often fail to modify autonomic dysregulation in response to present day experience (Fisher, 2019). Moreover, where the patient’s presenting symptomology is neurologically based, attempting to treat dysregulation through traditional talk-therapy approaches may elicit an increase in dysregulation rather than a resolution of the distress (McRae et al., 2012). The promise of cognitive training for mental health conditions lies in its ability to enhance cognition and community functioning, although more research is needed to understand its mechanisms and real-world applicability (Keshavan et al., 2014).

Cognitive Function Development Therapy (CFDT)—is a nonpharmaceutical, non-invasive treatment option for individuals aged 6 and above with a wide range of presenting symptoms (including, but not limited to anxiety, attentional problems, depression, psychosis, self-control challenges, and trauma-related conditions). Treatment decisions and outcome expectations of CFDT are based on objective assessment results mapped to five Primary Cognitive Functions (PCFs). These five PCFs include: Attentional Alerting, Attentional Orienting, Attentional Executive, Working Memory, and Encoded Memory (further explained in methods and Supplementary Data 1). Treatment is indirect (e.g., it does not seek to discover and ameliorate “root causes” of distress), thereby reducing the risk of treatment-related trauma or re-traumatization in clients. Rather, CFDT works by presenting targeted, interactive activities that engage and develop the client’s attention system, working memory, and encoded memory, reflected in PCF scores.

Cognitive performance can be improved through targeted training (Mandolesi et al., 2018; Thillier et al., 2023) and has been shown to have an effect on neurophysiological measures (Kariofillis et al., 2014). Therefore, it is important to explore the effects of novel cognitive training programs on neurophysiological measures. The brain vital sign framework (Hajra et al., 2016) utilizes portable and accessible electroencephalography (EEG) to extract well-established event-related potentials (ERPs) as objective neurophysiological indicators of cognitive information processing (Gawryluk and D’Arcy, 2010; Luck, 2014). Recent work has begun to use brain vital signs to track cognitive changes during rehabilitation (Fickling et al., 2020; Kirby et al., 2023), as well as attention training differences in healthy individuals (Smith et al., 2020). The brain vital signs framework elicits and records the N100, P300, and N400 in a portable, rapid and automated device that can be readily integrated at point-of-care (i.e., the NeuroCatch® Platform). Response latency (speed) and amplitude (size) are recorded for each of these three components. Slower and modulated responses, such as those for severe cognitive impairment in dementia (Ighalo et al., 2024) or after brain injury (Fickling et al., 2019b, 2021b; Fickling et al., 2021a), have been shown in previous work. In relation to mental health, the P300 attention related ERP component (Sutton et al., 1967) is commonly studied (Boudarene and Timsit-Berthier, 1997; Araki et al., 2005; Felmingham et al., 2002) and has been previously associated with changes in post-traumatic stress disorder (PTSD) related symptoms in a case study using the brain vital signs framework (Fickling et al., 2020).

The primary objective of this retrospective, observational study was to describe cognitive changes in a sample of children and adults undergoing CFDT and explore potential factors contributing to variation in cognitive changes. Our primary hypothesis was that individuals who have undergone CFDT would show improved cognitive measures as determined by analysis of the primary cognitive functions (PCFs). The secondary exploratory objective of this study was to evaluate changes in brain vital signs measures over time in a subset of program participants, to investigate the neurophysiological effect of CFDT. We anticipated that individuals would exhibit modulation of the P300 component over time in response to cognitive training.

## Methods

### Experimental paradigm

**Figure 1:**
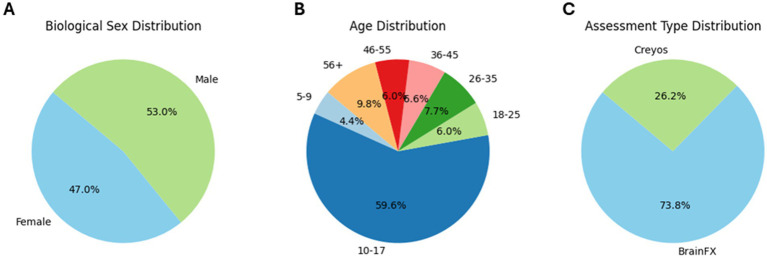
Pie chart of participant demographics: distribution of biological sex **(A)**, distribution of age bins **(B)**, and distribution of assessment type **(C)**.

Real world, de-identified data from 183 clients (Figure 1; demographics in Tables 1, 2) taking part in CFDT at a community mental health clinic (Polara Health, AZ, USA; CFD Services, AZ, USA) from 12/26/2019 through 5/13/2024 and overseen by the Cognitive Function Development Institute (CFDI), a nonprofit organization who developed CFDT, were included in the overall analysis. Clients selected for inclusion in the study were referred to CFDT (clinicians, school counselors, pediatricians, nurse practitioners, other clients, etc.) due to treatment resistance with traditional modalities (e.g., talk therapy) and neurological dysregulation (e.g., threat-related behavior without an observable, external environment threat present). For clinical intake purposes, client diagnoses include anxiety disorder, attention deficit hyperactivity disorder / attention deficit disorder, adjustment disorder, autism spectrum disorder, alcohol use disorder, bipolar disorder, developmental delay, disruptive mood dysregulation disorder, generalized anxiety disorder, impulse control disorder, major depressive disorder, obsessive-compulsive disorder, oppositional defiance disorder, opioid use disorder, persistent depressive disorder, PTSD, reactive attachment disorder, social anxiety disorder, traumatic brain injury, schizophrenia, and schizoaffective disorder. Note: clients may have multiple diagnoses. However, traditional diagnoses are not considered for CFDT purposes. Instead, CFDT treatment protocols are based on the most recent PCF assessment scores. Typical CFD therapy targets the two lowest PCFs with dynamically variable interactive engagements. For example, the therapist may present a pair of integers the client must add. Subsequently, the therapist repeatedly presents an integer and requires the client to add the therapist’s previous and currently presented numbers. If the client can do so, the therapist may simultaneously require the client to identify patterns of images in a densely populated, widely spaced visual field. The presented activities are increased in intensity incrementally until the client demonstrates fatigue, at which point the therapist will reset requirements for client responses. The pattern of fatigue and reset may be repeated two or three times in a typical session, often interlaced with a demonstration of client enjoyment without an easily identifiable reason. Each session is 1 h in duration. Client data were included in the analysis if the client had completed the same cognitive assessment (BFx or Creyos) before and after CFDT and if they received a minimum of 1,440 min of treatment, denoted as “Dosing” in the results (mean therapy duration between assessments [standard deviation]: 250.0 [135.2] days; range: 98–761 days). Final assessment results were taken from the client’s final cognitive assessment after completing CFDT (within 14 days of their final session). In addition, CFDI initiated a search for an objective neurophysiological assessment tool, and, recently started incorporating NeuroCatch as an exploratory tool. Therefore, data from a subset of 18 of these clients (demographics in Table 1) had been evaluated with NeuroCatch at two timepoints (mean therapy duration between scans [standard deviation]: 203.7 [93.0] days; range: 49–388 days).

**Table 1:** Descriptive demographics of study population.

Group	Number of participants	Biological sex	Mean age (standard deviation); [Range]
Total	183	86 females	23 (17) years; [7–83 years]
BrainFx (assessment type)	135	55 females	21 (14) years; [10–67 years]
Creyos (assessment type)	48	31 females	29 (23) years; [7–83 years]
NeuroCatch subset	18	11 females	30 (20) years; [10–61 years]

**Table 2:** Psychiatric diagnoses of study population.^†^

Psychiatric diagnosis	Percentage of study population
ADHD	32%
AjD	22%
PTSD	12%
BPD	10%
Other	46%

Diagnoses occurring in 10% or more of the population are listed. Other diagnoses include major depressive disorder (MDD), autism spectrum disorder (ASD), generalized anxiety disorder (GAD), alcohol use disorder (AUD), oppositional defiant disorder (ODD), disruptive mood dysregulation disorder (DMDD), opioid use disorder (OUD), schizoaffective disorder (SZA), schizophrenia (SCZ), and obsessive-compulsive disorder (OCD). ADHD, Attention-deficit/hyperactivity disorder; AjD, Adjustment disorder; PTSD, Post-traumatic stress disorder; BPD, Borderline personality disorder.

† Percentages do not sum to 100% as some clients have multiple diagnoses.

### Primary cognitive function scores

The 5 PCFs used in the CFDT framework are: Attentional Alerting (Callejas et al., 2004; Ex. notice (and name) things in the external or internal environment), Attentional Orienting (Callejas et al., 2004; Ex. select certain items from a larger field of items (select all the cards with a certain feature, like colour, from a messy pile of image cards)), Attentional Executive (Callejas et al., 2004; Ex. call out the characteristics of a card in a certain order amidst a wide or cluttered visual field), Working Memory (Ex. assign a number to a color and compute the value of the images), and Encoded Memory (Ex. recall information such as, patterns shown (then recreated)). Clients were objectively assessed using either the BrainFX® SCREEN (BFx) or the Creyos Health (Creyos) digital cognitive assessments at semi-regular intervals throughout their CFDT program. BFx is a function-focused assessment tool that addresses neurofunction through a digital interface on a tablet. Based on its more comprehensive predecessor the BrainFX® 360 (Searles et al., 2019), the BFx assessment is administered by a healthcare professional in 10–15 min via a touch tablet and has 7 domain-specific tasks. Similarly, the Creyos platform is used to administer 12 online cognitive tests to children, adolescents, and adults. The assessment results were then mapped to PCF scores. PCF mapping is a custom method developed by the CFDI to consolidate multiple scores from different assessment tools into a comprehensive, universal metric. PCF scores are normalized as an adjusted T-score (mean of 0 and standard deviation of 10) and are used to guide treatment decisions and outcome expectations. The mapping process for each of these assessments to PCF scores differs due to BFx and Creyos having a different number of output results and different standardization techniques. BFx was utilized for clients included in this study from December 2019 through June 2024. The CFDI started using Creyos as its primary assessment tool for new clients as of June 2023. The mapping process for each is outlined with more information on the 5 PCFs, as well as the BFx and Creyos tasks used, in Supplementary Data 1.

### EEG acquisition and processing

Brain vital signs were recorded using the NeuroCatch® Platform (Version 2.0, NeuroCatch Inc., Surrey, BC). Clients were fitted with a low-density EEG sensor cap (ANT Neuro Waveguard) with standard Ag/AgCl electrodes, and skin-electrode impedances were prepared to below 25 KOhms. Data were recorded from 3 midline electrodes (Fz, Cz, and Pz), with a ground electrode located at AFz, a reference electrode placed on the left earlobe, and a single electrooculogram (EOG) recorded from FPz. The scans took approximately 10 min in total, with brain vital sign testing for about 6 min. All clients received passive repeated auditory stimulation (ear insert headphone) of standard tones (80 dB) and random rare deviant tones (105 dB) ahead of basic spoken word pair primes that either matched or mismatched. The tones stimuli elicited the N100 and P300 and the word pair stimuli elicited the N400.

Recorded EEG traces were processed in Python. EEG data were filtered using a 0.1–20 Hz bandpass and 60 Hz notch filter. Ocular artifacts were corrected using an adaptive filter (He et al., 2004) with the EOG derived from the FPz channel. Stimulus-locked evoked epochs were extracted according to stimulus condition (i.e., standard/deviant tones, congruent/incongruent words). Epochs containing artifacts were rejected using an automated EEG signal-quality index (Fickling et al., 2019a). Artifact-free epochs were averaged for each stimulus condition to form representative ERP waveforms for each participant. The N100, P300, and N400 peaks were automatically detected and manually verified.

### Statistical analysis

To assess change in PCF scores, a mixed (*between-subject factors*: Sex [2: female and male] and Assessment Type [2: BFx and Creyos] and *within-subject factors*: Timepoint [2: pre- and post-treatment]) repeated measures multivariate analysis of covariance (RM-MANCOVA; *covariates:* [2: Age and Dosing]) was run. As a post-hoc analysis, all PCF scores were run in separate non-parametric Wilcoxon Signed-Rank tests due to the left skewedness of data. This is a non-parametric statistical test appropriate for non-normal data comparing the same group of subjects before and after an intervention (Woolson, 2005).

To further investigate potential factors affecting the change in PCF scores from pre- to post-treatment, a non-parametric Spearman’s rank correlation was run. Similar to the use of the Wilcoxon Signed-Rank test, non-parametric statistical tests like Spearman’s rank correlation are used for non-normally distributed data. The change in PCF scores were correlated against Age to better understand the effect this measure may be having on the change in PCF scores from pre- to post-treatment. All Wilcoxon Signed-Rank and Spearman’s rank correlation resulting *p*-values were *Bonferroni* corrected (5 scores, therefore *p*-values for each Wilcoxon Signed-Rank and Spearman’s rank correlation run are divided by 5). As Dosing was used to filter participants, this measure was further investigated in Supplementary Data 2.

As an exploratory sub-analysis, brain vital signs results were analyzed similarly to the PCF scores. The N100, P300, and N400 peaks were automatically detected and manually verified. A mixed (*between-subject factors*: Sex [2: female and male] and *within-subject factors*: Timepoint [2: scan 1 and scan 2]) repeated measures multivariate analysis of covariance (RM-MANCOVA; *covariates:* [1: Age]) was run. To ensure homogeneity of variance in this small sample size, Levene’s test of equality of error variance was performed for each brain vital signs component. Additionally, a post-hoc Pearson’s correlation analysis of P300 amplitude change (scan 2 P300 amplitude—scan 1 P300 amplitude) vs. Age was run.

## Results

### Primary cognitive function scores

**Figure 2:**
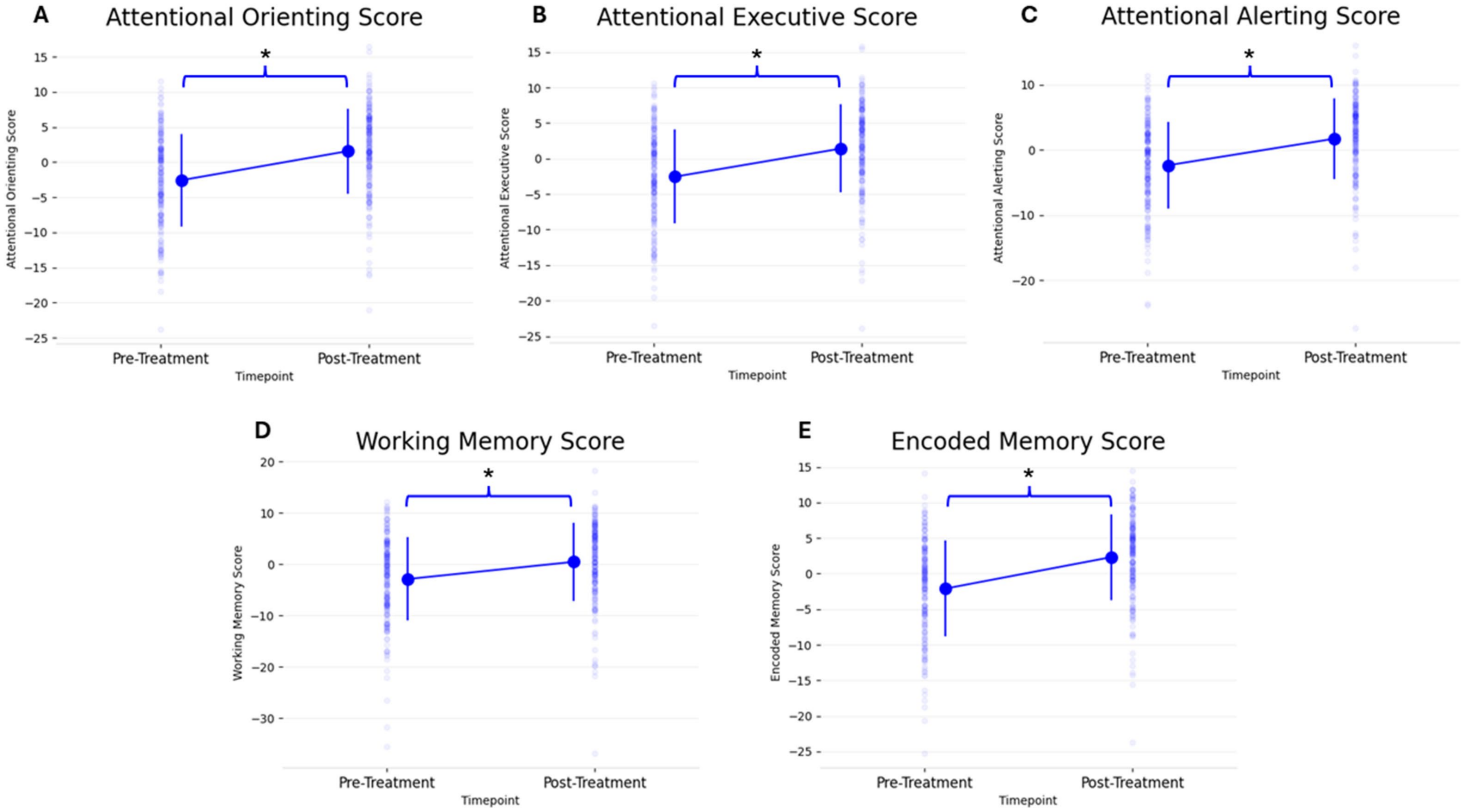
Change in all 5 PCF scores (**A**: Attentional Orienting, **B**: Attentional Executive, **C**: Attentional Alerting, **D**: Working Memory, **E**: Encoded Memory) from pre- to post-treatment. Individual scatter plots for each participant are shown faded in the background with group means denoted with large markers, including group standard deviation lines (*p < 0.05).

On a group level, the 5 PCF scores had a significant increase from pre- to post-treatment. Figure 2 and Table 3 shows the 5 different PCF scores from pre- to post-treatment. RM-MANCOVA results showed a multivariate effect of Timepoint [*F* (5, 173) = 7.087, *p* < 0.001, η_p_^2^ = 0.170], Age [*F* (5, 173) = 2.640, *p* = 0.025, η_p_^2^ = 0.071], and Assessment Type [*F* (5, 173) = 11.255, *p* < 0.001, η_p_^2^ = 0.245]. Additionally, results showed a significant interaction between Timepoint × Age [*F* (5, 173) = 3.696, *p* = 0.003, η_p_^2^ = 0.097]. The interaction between Timepoint × Assessment Type was not significant [*F* (5, 173) = 1.060, *p* = 0.384, η_p_^2^ = 0.030]. Further MANCOVA results are outlined in Table 4.

**Table 3:** Mean change of PCF measures (standard error).

Group	Attentional Alerting	Attentional Orienting	Attentional Executive	Working Memory	Encoded Memory
Total	4.491 (0.460)	4.656 (0.430)	4.468 (0.439)	3.968 (0.616)	4.928 (0.489)
BFx	3.973 (0.464)	3.986 (0.435)	3.793 (0.444)	3.216 (0.623)	4.261 (0.494)
Creyos	5.009 (0.835)	5.326 (0.782)	5.142 (0.798)	4.721 (1.119)	5.595 (0.888)

**Table 4:** MANCOVA multivariate results.

Multivariate Effect	*F*-value	*p*-value	Partial eta squared (η_p_^2^)
Timepoint	**7.087**	**<0.001**	**0.170**
Sex	0.789	0.559	0.022
Assessment Type	**11.255**	**<0.001**	**0.245**
Age	**2.640**	**0.025**	**0.071**
Dosing	1.204	0.310	0.034
Sex*Assessment Type	0.839	0.524	0.024
Timepoint*Assessment Type	1.060	0.384	0.030
Timepoint*Age	**3.696**	**0.003**	**0.097**
Timepoint*Dosing	0.807	0.546	0.023
Timepoint*Sex	0.361	0.875	0.010

Bold * - *p* < 0.05.

The PCF scores showed a left-skewed distribution, resulting in violation of a normal distribution. This is a common result in cognitive assessments (Saffari et al., 2024). Therefore, separate non-parametric Wilcoxon Signed-Rank tests were done to analyze each PCF score’s change from pre- to post-treatment. All PCF scores showed a significant change from pre- to post-treatment (Table 5).

**Table 5:** Wilcoxon signed-rank test results and Bonferroni adjusted *p*-values.

PCF Score	Wilcoxon Signed-Rank Statistic (W)	*p*-value (Bonferroni corrected)
Alerting	**2230.0**	<0.001*
Orienting	**1959.0**	<0.001*
Executive	**2188.5**	<0.001*
Working	**4014.5**	<0.001*
Encoded	**2201.0**	<0.001*

Bold * - *p* < 0.05.

### Brain vital signs results

**Figure 3:**
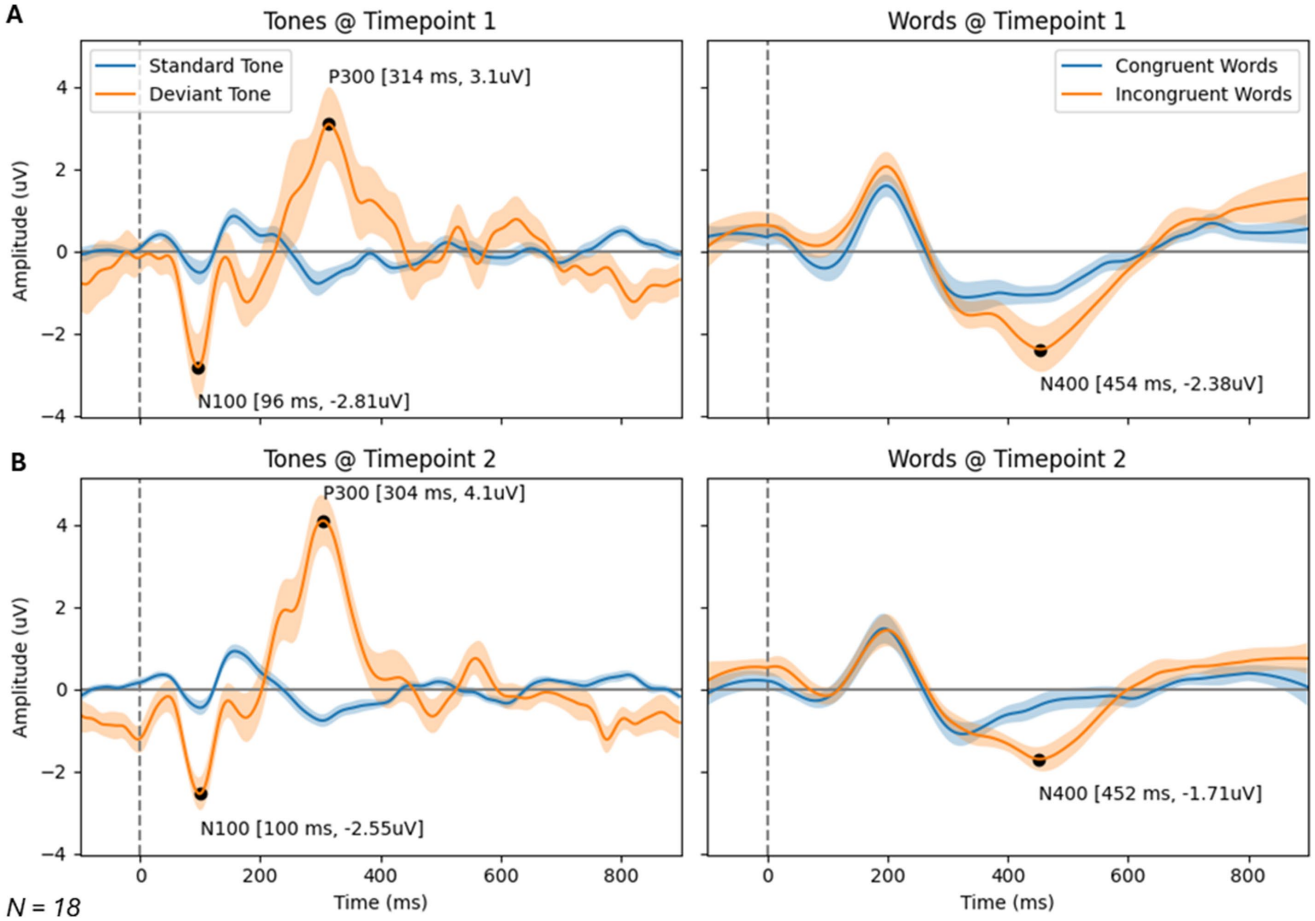
Brain vital signs waveform results at timepoint 1 **(A)** and 2 **(B)**. Waveforms elicited by standard and deviant tones (left) and congruent and incongruent word pairs (right). N100, P300, and N400 group average peaks are denoted with black dot, as well as a latency (ms) and amplitude (uV) label. A dotted line at time = 0 ms denotes stimuli event onset.

Brain vital signs group average waveforms at both timepoints showed a change in the P300 component (Figure 3). This change was confirmed with the RM-MANCOVA, showing a significant increase in P300 amplitude (Figure 3 and Table 6). However, no other brain vital signs components showed significant change (Table 6). Furthermore, all brain vital signs data passed Levene’s test of equality of error variances (all *p* > 0.05). Visually, brain vital signs waveforms seemed to have a higher group variability at scan 1 compared to scan 2 (shown by wider standard error shading in Figure 3, quantified in Supplementary Data 3).

**Table 6:** RM-MANCOVA univariate test results on the main effect of Timepoint.

Brain vital signs component	*F*-value	*p*-value	Partial eta squared (η_p_^2^)
N100 Latency	0.390	0.542	0.025
N100 Amplitude	0.003	0.958	0.000
P300 Latency	0.001	0.980	0.000
P300 Amplitude	**6.590**	0.021*	**0.305**
N400 Latency	0.729	0.407	0.046
N400 Amplitude	4.892	0.264	0.082

Bold * - *p* < 0.05.

Interestingly, the P300 amplitude showed a significant interaction of Timepoint × Age (Table 7). To further understand this, a post-hoc Pearson’s correlation between Age and P300 amplitude change showed a significant negative correlation (Pearson’s R: −0.590, *p* < 0.01).

**Table 7:** RM-MANCOVA univariate test results on the main interaction of Timepoint × Age.

Brain vital signs component	*F*-value	*p*-value	Partial eta squared (η_p_^2^)
N100 Latency	1.078	0.316	0.067
N100 Amplitude	0.146	0.708	0.010
P300 Latency	0.012	0.916	0.001
P300 Amplitude	**8.539**	0.011*	**0.363**
N400 Latency	3.449	0.083	0.187
N400 Amplitude	0.337	0.570	0.022

Bold * - *p* < 0.05.

### Correlations

Lastly, to further investigate the age effect noted in the primary RM-MANCOVA analyzing PCF scores, Spearman’s rank correlation was investigated. Correlation results showed a significant but weak negative correlation between the change in PCF scores and Age (Table 8; Spearman’s R range: −0.301 to −0.340).

**Table 8:** Spearman’s correlation results and Bonferroni adjusted *p*-values for change in PCF scores against Age.

PCF Score	Spearman’s Rho	*p-*value (Bonferroni corrected)
Alerting	**−0.340**	<0.001*
Orienting	**−0.329**	<0.001*
Executive	**−0.337**	<0.001*
Working	**−0.301**	<0.001*
Encoded	**−0.306**	<0.001*

Bold * - *p* < 0.05.

## Discussion

The current retrospective, observational study investigated real world data of cognitive performance changes in individuals taking part in CFDT. Specifically, the current study measured cognitive performance with PCF scores, derived from BFx and Creyos assessments before and after a minimum of 1,440 min of CFDT. The results suggest that CFDT improved cognitive performance measured with PCFs. Exploratory analyses suggest that CFDT may also lead to improvements in neurophysiological measures of cognition, measured as a P300 amplitude increase with the brain vital signs framework. Lastly, the results suggest that age may be contributing to variation in cognitive change with a weak, negative relationship between age and cognitive performance, as well as neurophysiological change.

### PCF scores

PCF scores, regardless of whether they were derived from BFx or Creyos assessments, improved with CFDT. Psychiatric disorders are commonly related to deficits in cognitive function, such as decreased attention, working memory, and disrupted social cognition (Trivedi, 2006; Wang Y. et al., 2022; Johnsen and Asbjørnsen, 2009; Burri et al., 2013; Kim et al., 2018). The improvement of mental health treatment seems to be a critical area of research as less than 20% of mental healthcare practitioners use or integrate measurement-based care in their practice (Lewis et al., 2018); thus, for those clients who can secure treatment, determining effectiveness of the treatment provided is dependent on highly subjective self-reported levels of patient distress rather than objective test results common with physical health care.

Digital cognitive performance assessment technology is shown to be useful in primary care settings and growing to better detect neuropsychological and cognitive deficits (Libon et al., 2023; Jannati et al., 2024; Banks et al., 2024; Brenkel et al., 2017; Levine et al., 2013). The cognitive tasks used in Creyos have been validated in several large-scale studies examining healthy controls and patient populations (Brenkel et al., 2017; Levine et al., 2013). Brenkl et al. compared cognitive impairment screening of tasks from Creyos with the commonly used Montreal Cognitive Assessment (MoCA). Authors found that the Creyos assessments provided more information that may be able to aid in differentiating levels of cognition in older adults (Brenkel et al., 2017). Cognitive testing also plays a role in cognitive rehabilitation, training, and even pharmacological development (Keefe et al., 2015; Wessels et al., 2021; Bahr et al., 2019; Preiss et al., 2013). Cognitive training offers behaviorally constrained cognitive or socio-affective learning events delivered in a scalable and reproducible manner to potentially improve neural system operations (Keshavan et al., 2014). By identifying specific areas of strength and weakness, cognitive tests can guide personalized programs aimed at improving specific cognitive functions (Preiss et al., 2013; Chen and Vinogradov, 2024). They allow for the monitoring of progress over time, providing feedback that can be used to adjust programs. It is important that cognitive training can affect other outcome variables besides cognitive function, and it implies the importance of better understanding cognitive improvements in mental health therapy (Kim et al., 2018).

Attention, encoded memory, and working memory are well-represented in the literature as individual topics of investigation. However, there exist challenges to leveraging the growing body of literature for clinical application in an integrative care setting. A form of mapping, such as with PCFs, provides an opportunity to track these cognitive functions across multiple cognitive tests (such as BFx and Creyos). It should be anticipated that networks governing a particular cognitive function (e.g., attention) will be coupled via edge interactions with networks that give rise to other cognitive functions (e.g., encoded memory; Bassett and Bullmore, 2017). It can be easily observed, for example, that cognitive functions operate on a coupled basis (Popp et al., 2024). However, the study of individual cognitive functions, as noted above, tends to obscure the possibility of such coupling, let alone interpret clinical applications for improving dysregulation. Conversely, the PCF mapping developed by the CFDI highlights the coupled nature of PCFs and provides actionable information to client-facing treatment providers regarding observable client behavior and therapeutic approaches to achieve the desired, targeted treatment. The PCF mapping method is noteworthy for two reasons. First, it provides an objective “line-in-the-sand” for each identified PCF against which improvements may be measured, as it also showed its robustness across different cognitive tests (no Timepoint*Assessment Type interaction noted). As an adjunct, therapists can readily identify functional areas to target for treatment based on the relative position of PCF scores (e.g., therapists target the two lowest functions for development). Second, graphing the obtained PCF scores produces a limited number of identifiable patterns with demonstratable behavioral explanatory or predictive ability. Therefore, demonstrating a positive change in PCF scores is an important initial step for CFDI to work towards a standardized level of evidence-based mental and cognitive healthcare.

### Brain vital signs

Previously, neuroimaging research focused on mental illness showed that cognitive training is associated with both structural and functional brain changes (Subramaniam et al., 2012; Kontis et al., 2013; McClure and Bickel, 2014; Li et al., 2022; González-Alemañy et al., 2024). More specifically to EEG neuroimaging research, the P300 oddball paradigm is one of the most heavily studied ERP components and is most associated with basic attention (Sutton et al., 1967). It is an indicator of the engagement of attentional resources to the stimuli, particularly related to the awareness of an individual’s surrounding environment. In relation to mental health, research suggests that individuals with PTSD exhibit abnormalities in information processing, reflected in ERP measures correlating with illness severity (Javanbakht et al., 2011). This includes a decreased P300 amplitude in groups with PTSD relative to controls (Boudarene and Timsit-Berthier, 1997; Araki et al., 2005; Felmingham et al., 2002). In a previous case study, brain vital signs have been sensitive to changes in the P300 ERP component during physical therapy paired with neuromodulation. These increases in P300 amplitude were paired with self-reported improvements in PTSD symptoms (Fickling et al., 2020). Based on previous research, the N100 and N400 ERP components show promise as biomarkers for psychiatric disorders. The primary finding being that schizophrenic patients show reduced N100 and N400 amplitudes (Jackson et al., 2014; Wang B. et al., 2022), with N400 abnormalities not being specific to schizophrenia, but also characterizing psychosis broadly (Jackson et al., 2014). It is interesting that these responses did not show changes in this subset. As a larger number of studies tend to focus on the P300, the N100 and N400 should be areas of future research on larger groups that are more organized by their diagnoses. The current study builds on this work, replicating the effect of increasing P300 amplitude alongside cognitive training therapy.

A disruption in brain physiology is suspected in several psychiatric disorders (Millan et al., 2012; Wang Y. et al., 2022; Paunova et al., 2023), which may be reflected in an increased neural variability (Menon, 2020; Månsson et al., 2022). Using functional magnetic resonance imaging (fMRI), Månsson et al. showed that task-based brain signal variability, historically considered a source of noise, was the strongest contributor to results of a treatment outcome-based prediction model, focused on measuring improvements in social anxiety. This variability measure also showed high test–retest reliability (Månsson et al., 2022). Speculatively, the variability in event-based neural responses measured with ERPs visually seen in the waveforms in Figure 3 (quantified in Supplementary Data 3) may be a potential area of interest for future biomarkers of psychiatric disorders or for treatment of psychiatric disorders.

### Correlations

Interestingly, there seems to be a significant, but weak, negative relationship between increasing age and the magnitude of change in PCF scores. This was both reflected in the significant Timepoint × Age interaction and the significant negative, but weak, Spearman’s rank correlation between each of the 5 PCF scores and age. While the ability to change and learn is kept throughout the lifespan, it is common for this ability to be somewhat lessened in older populations compared to younger populations (Pauwels et al., 2018). If the ability to improve cognitive performance is negatively associated with older age, it is critical for individuals to receive mental healthcare when problems first arise (Wass, 2015). Similar to the PCF result, there seems to be a significant relationship in the change in P300 amplitude and age. This was both reflected in the significant Timepoint × Age interaction and the significant Pearson’s correlation between the change in P300 amplitude and age. Future work may further clarify this with larger studies that are more evenly distributed across age ranges.

### Future work and limitations

The present analysis was a retrospective, observational analysis conducted with a diverse (i.e., range of ages, diagnosis types and complexities, and time in program), real world data set, limiting the interpretation of results. In relation to existing cognitive assessment tools used in CFDT (BFx and Creyos), PCF mapping was not affected by which tool was used (shown in the lack of a Timepoint × Assessment Type interaction effect). However, a large main effect of Assessment Type was present on an overall PCF score level. Therefore, while it seems like the change of PCF scores during therapy is not affected by the assessment used, the scores themselves may be on different scales depending on the assessment type used. The potential effect of assessment type used for PCF mapping should be better tested with more evenly weighted groups in future work or CFDT should dominantly focus on the use of a single assessment modality.

It was important to explore the possibility that brain vital signs framework’s objective neurophysiological measurements may be modulated by CFDT, to complement results from digital cognitive assessments. As this was an exploratory sub-analysis, future work can evaluate the effects on brain vital signs changes in larger samples of individuals taking part in CFDT. Additionally, future work may further clarify the connection between P300 amplitude change with larger studies that are more evenly distributed across age ranges. This can also include a correlation between brain vital signs measure changes and PCF score change to better understand the neurophysiological and cognitive performance connection. Future work to understand the connection between neurophysiology and cognitive performance may also look into deriving cognitive workload measures from continuous EEG during cognitive tasks (Mariani et al., 2023; Jia et al., 2021; Barzon et al., 2024). As can be seen from the PCF scores, CFDT emphasizes the importance of attention on primary cognitive function with three of the five PCFs being based in attention. The P300 is mainly associated with attention, therefore the most P300 change may also have the largest baseline attentional deficits, that are targeted in CFDT. As the current dataset is heavily weighted towards children (60% of PCF scores are from 10- to 17-year-olds), a more evenly distributed age range will also be useful for further analysis of the weak correlation between age and PCF and P300 changes.

Lastly, as clients were diagnosed with a range of mental health and/or cognitive concerns (such as PTSD, attention-deficit/hyperactivity disorder, bipolar disorder, etc.) and many clients had more than one diagnosis, the current study did not include ‘diagnosis’ as a predictive variable. Focusing on a particular clinical condition in the future (e.g., only adults with PTSD) may better clarify who CFDT is best suited for. Along with this, it is difficult to attribute the changes noted specifically to CFDT. Adding quality of life data and control groups in a prospective, randomized controlled study will improve the ability to draw more concrete conclusions about the program impact.

## Conclusion

The current retrospective study showed that clients taking part in CFDT for a minimum of 1,440 min of therapy improved cognitive performance as measured by PCFs. Given that mental illness is associated to cognitive deficits, it is noteworthy that the CFDT clients increased their cognitive performance during therapy. As an exploratory analysis, we also showed the feasibility and potential usefulness of an objective neurophysiological measure alongside digital cognitive performance measures. Analyses suggest that CFDT may also lead to improvements in neurophysiological measures of cognition, measured as an increase in P300 amplitude with the brain vital signs framework. Lastly, both PCF scores and P300 amplitudes showed a negative relationship between the magnitude of change and age. This may be an important aspect of therapy to investigate as it may stress the importance of seeking treatment as soon as possible to maximize the potential benefit or adjusting the dose of treatment for different ages. Overall, this retrospective, observational study is an important initial step for CFDI to work towards a standardized level of evidence-based mental and cognitive healthcare.

## Data Availability

The datasets presented in this article are not publicly available due to the sensitive nature of the information, which includes personal and health-related data of clinic clients, as well as ethical considerations and privacy agreements with the participants and their care facilities. Requests to access the datasets should be directed to Sonia Brodie, soniabrodie@healthtechconnex.com.
