# Supplementary material for: A retrospective, observational study of real-world clinical data from the Cognitive Function Development Therapy program
Source: Front Hum Neurosci. 2024 Dec 18;18:1508815. doi: 10.3389/fnhum.2024.1508815 (PMC11688245; doi:10.3389/fnhum.2024.1508815)
Supplement: Supplementary file 1 [file Data_Sheet_1.DOCX]

A retrospective, observational study of real-world clinical data from the Cognitive Function Development Therapy program

# Supplementary Material

***Supplemental Data 1: PCF Score Mapping***

*Further explanation of PCF scores:*

• **Attentional Alerting** (Callejas, Lupiáñez, and Tudela 2004) – which provides the capacity to prepare and sustain vigilance for noticing and processing stimuli. The stimuli may be equally noted from “bottom-up” or “top-down” channels. Bottom-up stimuli often come to the individual through the five senses or from encoded memories. It is identifiable with so-called “subconscious” information and, as should be expected, is frequently devoid of contextual (e.g., historical, geographical, etc.) or verbal information. Conversely, top-down stimuli include goals, choices, and so forth and thus be received through willful or effortful cognitive activity. Top-down information frequently contains rich contextual and verbal information. This therapeutic model recognizes that all stimuli, regardless of the environment to which they belong (e.g., the internal environment is sensed, for example, through the interoceptive senses or associated memory whereas the external environment is sensed through the five physical senses), must pass through or otherwise interact with the encoded memory system (Chun and Turk-Browne 2007). Consider, for example, going to a park. The external environment includes other individuals at the part, playground equipment, and so forth. In the same situation, the internal environment includes associated memories generated from experiencing the other people at the part and the autonomic nervous system response to said associated memories. The attentional alerting system filters / gates information from environmental stimuli on the basis of salience. Salience is determined by the ability of information to recruit alerting resources (Itti and Baldi 2009): Content (e.g., whether the information aligns with goal-oriented purposes, such as effortfully attending to this manuscript); Motion (e.g. moving objects could be a threat, a source of dopaminergic reward, or nothing consequential, the inherent uncertainty of initially noticed motion requires attentional capture); Signal Intensity (e.g., the brightest light, the loudest noise, the most offending odor, etc.; signals with greater intensity demand more attention than content or motion because of greater induced "free energy" (Whiteley 2009)); Potential Threat (e.g., Bayesian surprise, as opposed to Shannon surprise (Shannon 1948), which quantifies the improbability of a particular observation given a person's prior expectation (D’Alessandro et al. 2020), quantifies the magnitude of change to an individual's beliefs about the environment (Itti and Baldi 2009))

• **Attentional Orienting** (Callejas, Lupiáñez, and Tudela 2004) – provides the ability to select relevant information from the plethora of all noticed information, often with an associated selection of sensory input or stimuli location. Whereas the alerting network enables the info to notice stimuli, orienting sorts the noticed stimuli based on relevance. Example: select certain items from a larger field of items (select all the cards with a certain feature, like colour, from a messy pile of image cards). The attentional orienting function filters salient information on the basis of relevance. Relevance (as opposed to irrelevance or distraction) is determined by the individual's prior belief about the environment(s) on a task-related basis (D’Alessandro et al. 2020). Filtering relevant information from that which is irrelevant is a Bayesian endogenous attentional process (Mirza et al. 2019). For example, an individual is exposed to many road signs while highway driving. Suppose the individual is looking for indications of a desired off-ramp. In that case, it is reasonable to assume the individual’s prior belief about the road environment includes a belief that the off-ramp will be timely indicated. The driver will undoubtedly see other signs on the road while watching for the off-ramp indication. However, most of the signs are not relevant to the task of locating and taking the desired off-ramp. The driver will thus filter them out as distracting or irrelevant.

• **Attentional Executive** (Callejas, Lupiáñez, and Tudela 2004) – allows for resolving conflicts in information selection by prioritizing relevant information (Rueda et al. 2015). The determination of informational priority follows closely the determination of salience on a task-by-task basis (Lenartowicz et al. 2018). The therapeutic framework supports the hypothesis that synthesizing top-down and bottom-up motivational processes substantively contributes to a supramodal determination and application of informational priority (Spagna, Mackie, and Fan 2015). Example: call out the characteristics of a card in a certain order amidst a wide or cluttered visual field.

• **Working Memory** – defined as the small amount of information that can be held in an especially accessible state and used in cognitive tasks. Some research suggests that items stored in working memory are encoded as gamma frequency bursts held within a coupled theta frequency (Lundqvist et al. 2016; Moran et al. 2010; Ward 2003). Therefore, information stored in working memory only remains if it remains the object of the attentional system (Moran et al. 2010). Example: Assign a number to a color and compute the value of the images.

• **Encoded Memory** –Encoded memory is the record of information and experiences, including sensory and behavioral, that is encoded and stored for later retrieval by various neural networks within the brain and is critical for day-to-day functioning. Encoding is the process of moving information from our short-term, or working memory, into our long-term memory and results from short or long-term neural remodeling (Stacho and Manahan-Vaughan 2022). Example: Recall information such as patterns shown (then recreated).

*Further explanation of PCF score mapping:*

BFx assessment’s provided scores are mapped to PCF adjusted T-Scores (adjusted T-Scores have a mean of 0 and standard deviation of 10). Each of the 7 BFx scores is used and weighted differently to calculate each of the 5 resulting PCF scores. This is based on the tasks BFx uses to calculate their 7 scores. The CFDI postulates that primary cognitive functions operate on a coupled basis. As such, each PFC may be targeted for engagement and development. However, isolating a targeted PCF from other PCF expressions is impossible. For example, adding two numbers depends heavily upon and targets the PCF Working Memory. Completing the task also requires the expression of Encoded Memory (to recall what each number is and means) and attentional PCFs (to maintain the information held in Working Memory while excluding distracting information). Thus, some tasks used to calculate abstract reasoning in BFx engage both attentional alerting and attentional orientation, though in differing amounts. BFx has seven domain-specific tasks. The domain tasks include:

1. Abstract reasoning
2. Constructive ability
3. Visual spatial awareness
4. Problem solving
5. Route finding
6. Prioritizing
7. Divided attention

Creyos calculates assessment results as standard scores (mean of 100 and standard deviation of 15). Creyos’ standard scores are then further processed by a regression to the mean to produce their outputted adjusted standard scores. Therefore, PCF mapping of Creyos scores begins with a rollback of Creyos' regressed scores to estimate the earned standard score. The rollback is calculated using best-fit 6^th^-degree polynomial trendlines as the tightest fit to the distribution of the data. Next, rolled-back Creyos scores are mapped to the 5 PCF scores by computing an adjusted T-score (mean of 0 and standard deviation of 10) determined as a multivariate best-fit to comparative BFx-based PCF scores (e.g., setting the 12 Creyos subtest scores at the standard score mean should result in each of the 5 PCF scores achieving the PCF mean value). The following 12 Creyos tests were utilized by the CFDI:

1. Short Term Memory
   1. Monkey Ladder
   2. Spatial Span
   3. Paired Associate
   4. Token Search
2. Reasoning
   1. Odd One Out
   2. Spatial Planning
   3. Rotations
   4. Polygons
3. Concentration
   1. Double Trouble
   2. Feature Match
4. Verbal Ability
   1. Digit Span
   2. Grammatical Reasoning

***Supplemental Data 2: Dosing Analysis***

As Dosing was a parameter used when filtering participants in the current analysis (Dosing demographics in Supplemental Table 1), it was further investigated as a potential factor affecting the change in PCF scores from pre- to post-treatment. The previously reported RM-MANCOVA (Table 3) did not show a significant Timepoint × Dosing interaction [*F* (5, 173) = 0.807, *p* = 0.546, η_p_^2^ = 0.023]. A non-parametric Spearman’s rank correlation was run. The change in PCF scores were correlated against Dosing. All Spearman’s rank correlation resulting *p*-values were *Bonferroni* corrected (5 scores, therefore *p*-values for each Spearman’s rank correlation run are divided by 5). The changes in PCF scores were not correlated with Dosing (Supplemental Table 2: *p-*values ranged from 0.265 to 1.0, *Bonferroni* corrected).

**Supplemental Table 1:** Dosing demographics of study population.

| Group | *Dosing minutes (standard deviation); [Range]* |
| --- | --- |
| Total | 2656.5 (1323.4); [1440 – 9727] |
| BrainFx (assessment type) | 2959.9 (1409.3); [1446 – 9727] |
| Creyos (assessment type) | 1803.0 (322.8); [1440 – 2836] |

**Supplemental Table 2:** Spearman’s correlation results and Bonferroni adjusted *p*-values for change in PCF scores against Dosing (*** - *p < 0.05***)**.**

| PCF Score | *Spearman’s Rho* | *p-value (Bonferroni corrected)* |
| --- | --- | --- |
| Alerting | 0.113 | 0.633 |
| Orienting | 0.143 | 0.265 |
| Executive | 0.109 | 0.713 |
| Working | 0.033 | 1.0 |
| Encoded | 0.129 | 0.403 |

***Supplemental Data 3: Standard Error Quantified***

The mean of the standard error shading shown in Figure 3 was determined to quantify the visual decreasing of ERP variability shown.

**Supplemental Table 3:** Mean of standard error across the group averaged waveforms in Figure 3**.**

| Waveform | *Timepoint 1 Standard Error Mean* | *Timepoint 2 Standard Error Mean* |
| --- | --- | --- |
| Deviant Waveform | 0.61 | 0.44 |
| Incongruent Waveform | 0.37 | 0.27 |
